# Open surgical approach to fractures of the mandibular condyle: surgical technique and associated complications

**DOI:** 10.55730/1300-0144.5887

**Published:** 2024-05-22

**Authors:** Cenk DEMİRDÖVER, Alper GEYİK

**Affiliations:** Department of Plastic, Reconstructive, and Aesthetic Surgery, Faculty of Medicine, Dokuz Eylül University, İzmir, Turkiye

**Keywords:** Maxillofacial surgery, mandibular fractures, mandibular condyle, internal fracture fixation, complications

## Abstract

**Background/aim:**

This study evaluates anatomical reduction and rigid internal fixation of mandibular condyle fractures using the preauricular retroparotid approach. It also discusses advantages, deficiencies, and associated complications of the technique.

**Materials and methods:**

This retrospective study reviewed the medical records of a total of 52 mandibular condyle fractures from 42 patients who were treated with open surgery using the preauricular retroparotid approach between January 2019 and January 2024. Preoperative and postoperative assessments included measurements of mouth opening (maximum interincisal distance), vertical mandibular movement, and facial paralysis. Moreover, the Vancouver Scar Scale (VSS) was used to evaluate scar quality at the surgical site. Descriptive statistics were used to summarize patient demographics, preoperative findings, and postoperative outcomes.

**Results:**

Anterior open bite was the most common finding, detected in 83% of the patients before surgery. The mean mouth opening of the patients increased significantly from 29 ± 4.94 mm to 37.76 ± 2.12 mm. Vertical mandibular movement exceeding 4 cm was a finding in more than half (52.3%) of the patients. The mean VSS score, indicating scar quality, was 1.64 ± 0.70, suggesting overall good cosmetic outcomes. Plate breakage in two patients was noted as a complication during follow-up.

**Conclusion:**

Several surgical techniques have been described for mandibular condyle fractures, each with its own benefits and limitations.

## 1. Introduction

The mandibular condyle is the region most susceptible to fracture because of the forces transmitted during trauma [1,2]. The majority of etiologies entail traffic accidents, assaults, and falls [3]. Despite the standard treatment of other mandible fractures being open reduction and internal fixation (ORIF), no such consensus has been reached for the condylar region. Both clinical and radiological findings are essential for determining the surgical approach [4].

A myriad of classification systems have been developed to guide surgeons’ preferences. Lindahl’s classification system is the most utilized in the literature and includes the level of fracture, level of dislocation, and position of the condylar head relative to the glenoid fossa (Table 1) [5]. For decades, the only treatment options for mandibular condylar fractures were closed reduction and maxillomandibular fixation (MMF) with arch bars; however, in the literature, there has been a recent tendency toward ORIF, except in cases of condylar head and childhood fractures [6]. The application of miniplates is the most widely accepted osteosynthesis technique of open fixation [7]. Indications for ORIF are well documented in the guidelines (Table 2) [8]. Ramus height instability that affects occlusion and failure of closed reduction for condylar displacement are two absolute indications for open reduction [9].

Many surgical techniques have been introduced for the reduction of mandibular condylar fractures. Complications associated with ORIF are related to the surgical site and technique. Facial asymmetry, chronic pain, malocclusion, and condylar resorption are the most frequent site-related complications. In contrast, hemorrhage, seroma, infection, fistula formation, temporary or permanent nerve palsy, scarring, repair material breakages, or losses are technique-related complications [10].

The purpose of this study is to describe our surgical treatment of open condyle fractures and present our functional outcomes.

## 2. Materials and methods

### 2.1. Diagnosis

This study was approved by the relevant ethics committee (Approval No: 2024/05-14). Most of the patients were examined in the emergency service. The patients were questioned about concomitant diseases, medications, smoking, and alcohol consumption. A physical examination was then performed, beginning with the inspection of the trauma site. Missing teeth, dental status, laceration, bruises, and ecchymosis were recorded. Visual acuity and facial and sensory nerves were examined. Any clinical signs of condylar fracture such as preauricular pain and tenderness on palpation, trismus, malocclusion, and deviation during mouth opening were evaluated. An orthopantomogram and thin-slice computed tomography (CT) scan in 3D and in the axial and coronal planes were obtained before and after the operation. The CT scans precisely outlined the site and extension of the fracture and the degree and direction of displacement. Before the operation, each patient was provided with a detailed explanation of all aspects of the procedure. They then signed informed consent forms, confirming their acceptance of the open reduction. Photos with a mouth-opener were taken both before and after surgery.

### 2.2. Patients

A retrospective study was carried out from January 2019 to January 2024, initially involving 162 patients, with a total of 219 fractures of the mandibular condyle treated. Data on demographics, treatment modalities, and outcomes were retrieved from our clinic’s medical records system. Forty-two patients (26%, 52 fractures) were treated with ORIF and were included in the study. The study was approved by the local institutional ethics committee and conducted in accordance with the principles of the Declaration of Helsinki, and all patients provided written informed consent prior to enrollment. Twenty-nine patients (69%) were male and 13 patients (31%) were female (Table 3). The ages of the patients ranged from 19 to 55 years and the average age was 28.9 years. Ten patients (24%) had bilateral condylar fractures and 32 patients (76%) had unilateral condylar fractures. Thirty patients had additional fractures in different anatomical regions of the mandible. Falls were the most common etiological factor. The time between injury and operation ranged from 3 to 26 days and the average was 13 days. All patients had an extraoral incision, a preauricular short scar, and a retroparotid approach. The surgeon decided to perform ORIF according to patients’ preferences, defined indications, and clinical and radiological findings.

### 2.3. Surgical technique

**Figure 1:**
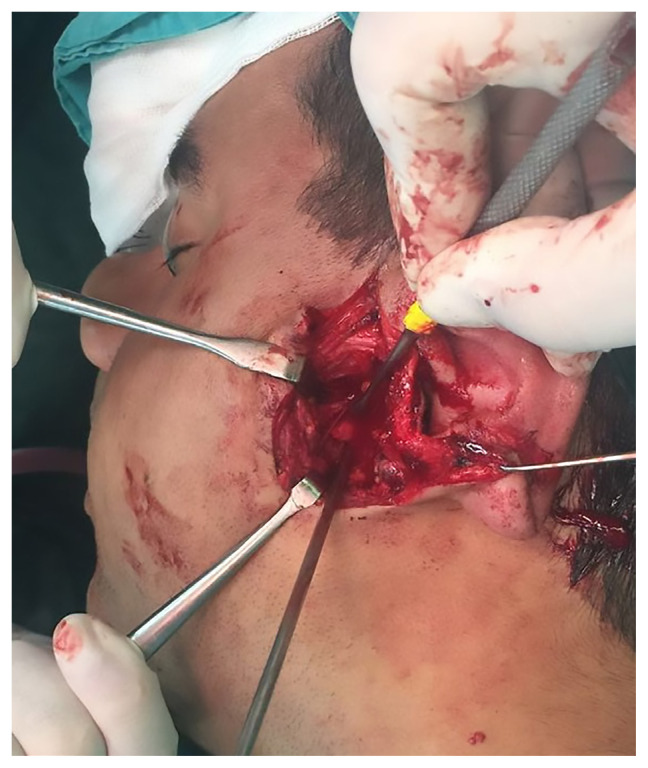
Exposure of anatomic structures before dissecting the condyle and the fracture site.

**Figure 2:**
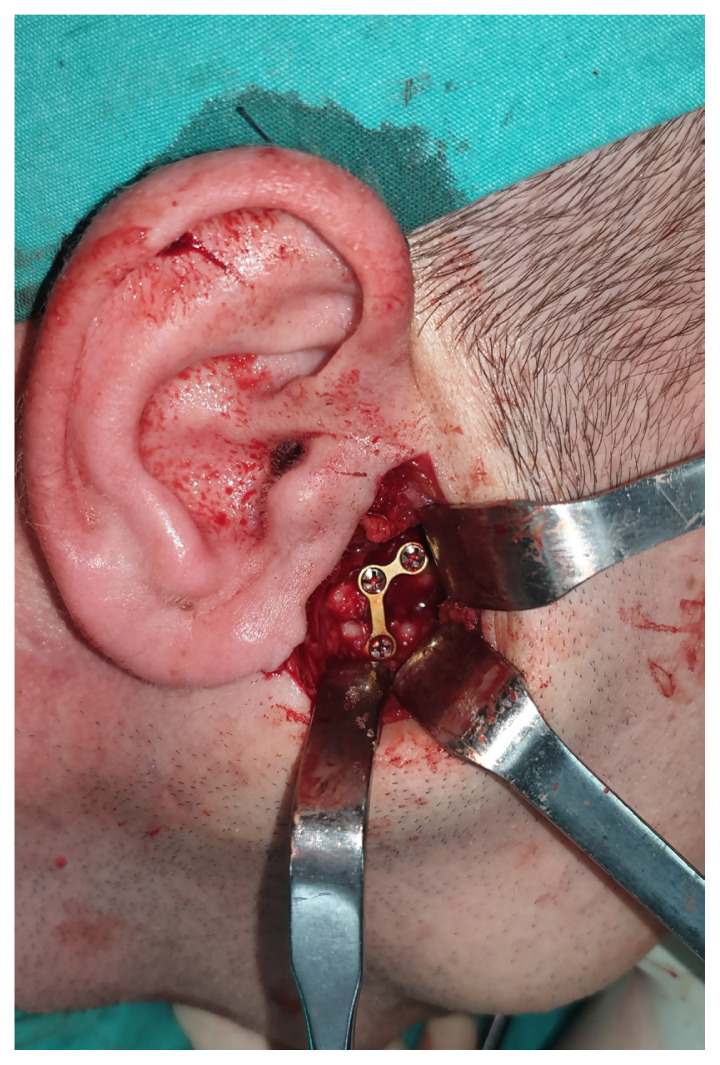
Fixation of the condylar neck fracture with an L-shaped miniplate.

**Figure 3:**
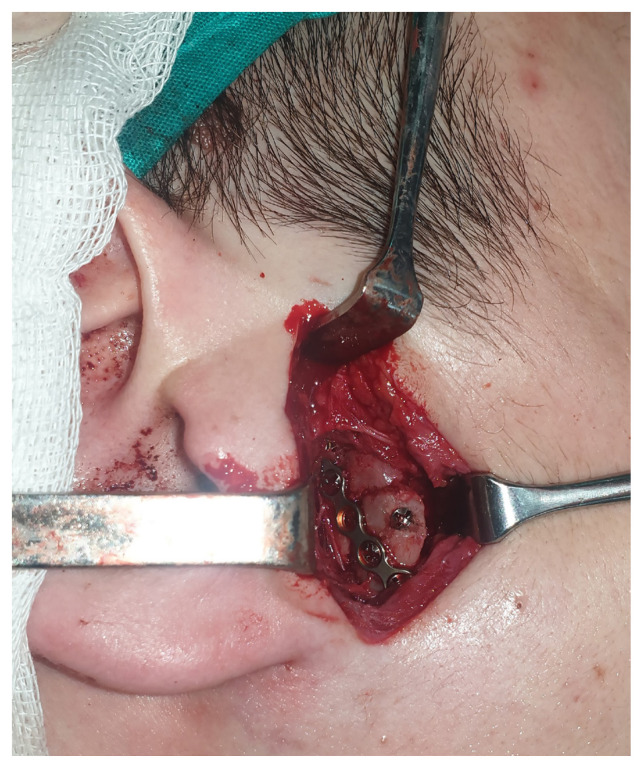
Fixation of the subcondylar fracture with a bent miniplate.

**Figure 4:**
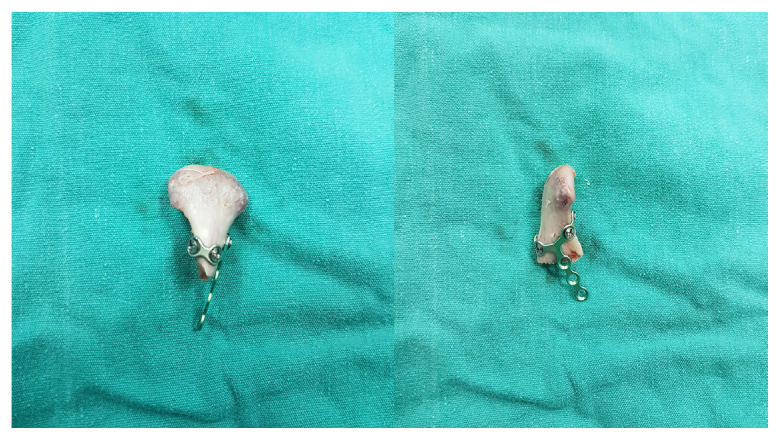
Extracorporeal fixation of the condyle fracture.

After nasotracheal intubation, skin markings were made for a preauricular short scar incision. The surgery was performed in accordance with the following steps, which can be customized based on the type of fracture:

- The preauricular-retroparotid approach was performed through a skin incision of 3–3.5 cm that extended from the level of the arcus zygomaticus to the intertragal notch.
- The position of the skin incision was posterior to the parotid gland. Dissection was carried out directly to the deeper layers. The risk of jeopardizing the temporal branch of the facial nerve would be higher if the incision proceeded cranially or toward the anterior side.
- Gentle retraction and the use of reconstituted adrenalin for hemostasis were two key factors in preventing neuropraxia.
- Below the zygomatic arch, the subcondylar region was palpated to ensure that the anatomic plane was correct. Palpation of the bone with the fingertips was used for guidance (Figure 1).
- With the assistance of retractors, the superior fibers of the masseter muscle were dissected and a window was opened to the periosteum.
- Attention was given to the maxillary artery and pterygoid plexus while dissecting the periosteum.
- As shown in Figures 2 and 3, the fracture side was visualized. Dislocation at the fracture level and the position of the condylar head were evaluated. A bone clamp was used for the reduction of the angulation and dislocation. Pulling the angle of the mandible downwards intraorally helped to increase the visual field. As shown in Figure 4, we would have opted for the extracorporeal approach if the intracorporeal reduction had been insufficient.
- The disadvantage of the short incision was restricted manipulation of the fractured segment; therefore, we sometimes preferred extracorporeal fixation to obtain proper alignment.
- The edges of a double Y-plate were bent inward to cover the bone at each aspect. According to the extent of fracture, an L- or Z-plate could also be used. MMF was used for most of the patients.
- While performing the extracorporeal approach, replacement of the condylar head with the plate in anatomic position could prove troublesome; effective intraoral retraction was therefore crucial.
- After proper reduction, the subcutaneous tissue was repaired with 3.0 and 4.0 round polyglactin and the skin was sutured with 5.0 poliglecaprone. Drains were used as necessary.

### 2.4. Postoperative care and follow-up

**Figure 5:**
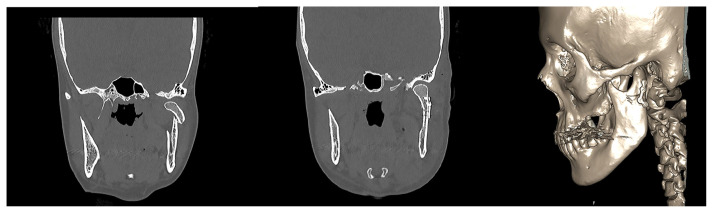
Postoperative CT scan of a condyle fracture after fixation.

**Figure 6:**
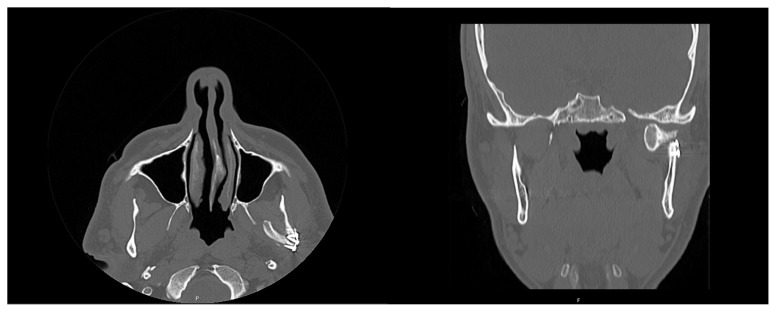
Postoperative view of plate failure in a condyle fracture.

Antibiotics and nonsteroidal antiinflammatory drugs were prescribed for 1 week. Patients were allowed to drink water after the first postoperative day. Oral intake was then increased gradually according to patient compliance. The patient’s head was stabilized and prevented from turning to the operated site. After extraction of the elastics, we made a stair-like instrument from a tongue depressor, which was adjustable and had a length of 4–5 cm. We trained the patients to perform a set of exercises. Patients inserted a part of the tongue depressor once every hour and were asked to open their mouths until they felt pain; in this way, we encouraged the gradual recovery of the normal range of jaw movement. The presence of concomitant fractures treated with an intraoral approach was a contradiction for this treatment protocol. As shown in Figure 5, control CT imaging was performed in the first few days after surgery. We evaluated postoperative occlusion by asking the patients whether they perceived their occlusion to be the same as that experienced before the trauma. Mouth opening, dental occlusion, facial nerve functionality according to the House–Brackman Facial Nerve Grading System [11], Helkimo’s Clinical Dysfunction Index (evaluating impaired range of motion and tenderness to palpation of the temporomandibular joint [TMJ]) [12,13], and the Vancouver Scar Scale (VSS) [14] were evaluated 6 months after the operation. Subsequently, patients were followed for a maximum of 14 months from the start of the study, with an average follow-up time of 8.2 months. Wound site infection, plate fracture (as shown in Figure 6), and permanent paralysis of the facial nerve were taken into consideration as complications.

## 3. Results

Preoperative mouth opening was limited, with an average of 29 mm (range: 15–47 mm). Following surgery, patients experienced significant improvement in mouth opening to an average of 37.76 mm (range: 34–42 mm) (p < 0.001).

Two patients (4.8%) had normal occlusion before the operation. Out of the 42 patients in the study group, 35 patients (83%) had an anterior open bite and 9 patients (21.4%) had a lingual or buccal crossbite. After the operation, two plates fractured (4.8%) and malunion was determined; these patients still had an open bite and therefore underwent revision surgery. Eight patients (19%) were treated with the extracorporeal approach.

The House–Brackman Facial Nerve Grading System was used to evaluate preoperative and postoperative facial nerve function. All patients except one regained normal facial nerve function (grade I) within 6 months after surgery. In the early postoperative period, a temporary decline in facial nerve function was observed, with 31% of patients experiencing grade II weakness and 21.4% experiencing grade III weakness.

Vertical mandibular movement was ≥40 mm in 22 patients (52.3%) and 30–40 mm in 20 patients (47.7%). Three patients (7.1%) had a slight deviation and one patient (2.4%) had a locked TMJ. Two patients (4.8%) had tenderness on palpation both laterally and posteriorly.

Evaluations using the VSS revealed good cosmetic outcomes, with an average score of 1.64 ± 0.70 (ranging from 0 to 4 points, with 0 indicating the best cosmetic outcome). Notably, nine patients (21.4%) achieved a perfect score of 0, indicating minimal to no scarring.

Bleeding, hematoma, seroma, Frey syndrome, and parotid fistula were not encountered as complications during the period of this study.

## 4. Discussion

Closed and open surgical approaches are two treatment modalities for mandibular condyle fractures. Despite the well-defined indications, consensus has not been reached regarding which modality produces the best functional results. The fracture classification, imaging modality, and surgical intervention differ among clinics worldwide and are largely based on the surgeon’s preferences and experience [15,16].

Nondisplaced condylar head fractures and most pediatric fractures are treated with a conservative or closed approach. For edentulous patients, when the condyle is neither dislocated nor displaced, conservative treatment yields satisfactory results. However, if there is a decrease in mandibular height, ORIF remains the preferred option, although patients’ health conditions may impose restrictions [17]. Apart from these limited indications, the literature indicates that ORIF is the only reduction method that can precisely realign the fractured segments [18,19].

Due to developments in the available hardware systems and increasing surgical experience, ORIF has become a reasonable treatment option. However, Ellis et al. emphasized that the potential risks of ORIF must be evaluated carefully against its potential benefits [20].

Among the three major skin incisions used in the extraoral approach [21], we prefer the preauricular incision; however, the incision we described in this study differed from that in the literature in that it was short and more anteriorly positioned [22]. Algan et al. presented an approach similar to ours; they made an additional preauricular incision to reach the fracture in the condylar region [23]. However, we always used an uninterrupted incision of at least 3 cm because the preferred approach should enable the surgeon to view the fracture site. We believe that reduction, manipulation of the hardware, and screw fixation are more difficult when the incision is shorter than 3 cm in length.

**Figure 7:**
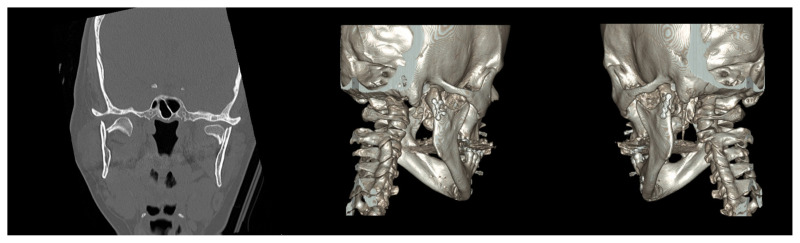
Three-dimensional image of a patient after fixation for condyle fracture.

Various fixation techniques have been published, including screws and both single and double miniplates [23]. Clinical studies have indicated that single-plate fixation cannot provide adequate rigidity and stability, while two miniplates comply with the principles of osteosynthesis and provide better functional outcomes [24–26]. Furthermore, a plate design factor was established to calculate fixation rigidity [27]. We utilized a 2.0 titanium mini double Y-plate with six holes for most patients and bent the plate inward to cover all sides before the placement. According to the literature, manual bending can change the physical properties of the osteosynthesis [28]. In addition, the osteosynthesis was much more stable when six or seven screws were inserted to fix the plate. In a similar method, we used 2.0 system plates and inserted six screws; however, we preferred the single-plate method as shown in Figure 7. Some of the current literature supports the claim that double plating yields superior results compared to single plating [29]. It also appears that using a single miniplate is associated with unstable osteosynthesis and displacement along the fracture line. Therefore, some authors strongly recommend the use of two miniplates for fixation [30,31]. Our rates of complications such as plate failure and screw loosening were lower than those indicated in the literature [32]. The utilization of a minidynamic compression plate and plate bending are among the suggestions for practitioners who intend to use a single plate [33]. Technological developments, such as custom-made approaches and various 3D plate types, have helped overcome the problems associated with the single-plate method [34–36]. Additionally, preauricular incisions are not suitable for fixation with two plates due to the limited surgical site.

A metaanalysis reported that the mean proportion of cases with hardware failures was more than 6.5%. It was further stated that combining the fixation method with MMF had a moderate effect on the occurrence of hardware failure [24]. In another metaanalysis, the application of MMF during surgery occurred in 34.3% of the reviewed studies; however, this information was not reported in 60% of the studies. Most of the studies highlighted the use of MMF, but it could be argued that the main advantage of open treatment is that MMF should not be required [21]. We applied MMF for 88% of our patients. Our hardware failure rate was 4.7%, slightly lower than that of other studies, but the average follow-up period of our study was 8.2 months.

Facial nerve palsy is a devastating complication and is considered a potential reason to avoid open surgery [20]. The preauricular approach is thought to present a higher risk of facial nerve injury, hypoesthesia, hematoma, and hypertrophic scars [37]. On the contrary, a recent review implied that branches of the facial nerve are not even in the dissected area with this approach due to the high location of the incision. It was also indicated that it is an inadequate approach for the reduction of subcondylar fractures. In addition, the incidence of facial nerve damage with the preauricular approach has been reported to be 3%–48% [38]. In other reported studies, approaches were classified as transparotid or nontransparotid, and temporary facial paralysis was encountered in 42.4% and 34.5% of cases, respectively. While a recent review [39] suggested an 11.8% risk of permanent facial paralysis associated with the transparotid approach, another study [40] reached different conclusions.

According to another metaanalysis, the perceived risk of transient weakness was 4% for the anteroparotid approach and 8% for the transparotid approach [37]. The retromandibular transparotid approach was directly correlated with nerve dysfunction [38].

Our underlying reason for using the preauricular retroparotid approach was to avoid transecting the gland. Although we did not encounter or dissect facial nerve branches, we detected grade II and III facial paralysis. This highlights the importance of patient counseling regarding potential temporary facial nerve weakness following surgery. Only one patient (2.4%) had permanent paralysis of the temporal and marginal mandibular branches of the facial nerve, but this patient was admitted to an intensive care unit for 55 days before the operation and had concomitant facial and cranial fractures. As a result, our transient facial nerve injury rate is higher than that of the literature [23,27,41]; however, none of those previous studies evaluated nerve function with a grading scale. In another study, grade II facial weakness was encountered in 40% of the patients and was observed 6 months after surgery. Most patients with facial weakness were treated using the retromandibular retroparotid approach. The authors mentioned that none of the patients showed permanent damage to the facial nerve [42]. We suggest that, in cases where techniques for short scars are preferred, excessive traction, manipulation, and electrocauterization of the vessels adjacent to the facial nerve can cause neuropraxia and loss of nerve function [39–43].

The VSS was developed to evaluate the adequacy of treatment and assess outcomes in burn patients [44]. We applied this scale to postoperative preauricular scars. Ten patients (23.8%) reported a score of 3 points and one patient reported 4 points. In the literature, the risk of undesirable scarring was present in ≥2.4% of cases [21]. In our study, the occurrence of poor scar quality was lower than that reported in the literature [38]. It has been noted that erroneous planning of the preauricular incision can lead to visible preauricular lines, an unnatural tragal appearance, and loss of earlobe definition [45]. Despite the association of preauricular incisions with poor scarring [21], a shorter incision and subcuticular repair can resolve this issue.

After the operation, three patients (7.1%) had deviation with mouth opening and one patient (2.4%) had ankylosis. Studies have reported 72.7%–100% of patients having no occlusal disturbances at the end of the follow-up period. Disocclusion is a significant problem, especially following condyle fractures, and its treatment remains challenging. Various types of osteotomies related to orthognathic surgery can be performed. The time interval between the trauma and disocclusion treatment is also crucial for determining the appropriate surgical intervention [46]. Our results are consistent with the literature, in which the presence of malocclusion was reported to range from 0% to 27.3%. Measurements of mouth opening differed between studies. We considered mouth opening of more than 3 cm as a “good” outcome. Limited activity has been reported in 0%–27.3% of cases with a reduced range of motion of the mandible in 0%–42.1% of cases [47]. In contrast to the literature, we observed one case of ankylosis during the study period. No surgical complications were observed, including hematoma, wound infection, Frey syndrome, or fistula.

This study’s retrospective design limits causal inferences. The moderate sample size and relatively short follow-up period warrant caution while generalizing the findings. Additionally, the study did not assess patient-reported outcomes such as pain or satisfaction.

## 5. Conclusion

Various techniques for treating mandibular condyle fractures have been outlined in different studies. Our surgical approach addresses the limitations of traditional techniques, such as the difficulty of inserting two miniplates and the risk of permanent facial nerve injury. However, further studies with larger prospective designs are needed for confirmation.

## Figures and Tables

**Table 1:** Lindahl’s classification system.

Fracture level		Dislocation at fracture level of condyle and subcondyle	Position of condylar head to articular fossa
Head	Vertical	Angulation medial override	No displacement
	Horizontal		
	Compression		
Neck		Angulation lateral override	Slight displacement
Subcondylar		Angulation without override	Moderate displacement
		Fissure	Dislocation

**Table 2:** Indications for open reduction.v

Displacement into middle cranial fossa
Tympanic plate injury
Impossibility of obtaining adequate occlusion
Lateral extracapsular displacement
Invasion by foreign body
Failure to obtain segment contact because of intervening soft tissue
Blocked mandibular opening
Facial nerve paresis secondary to initial injury
Contraindicated intermaxillary fixation
Open wounds from initial injury

**Table 3:** Demographic analysis of patients and fractures.

Age, years		28.95 ± 12.06

Sex	Male	29 (69%)
	Female	13 (31%)

Etiology	Fall	18 (42.8%)
	Traffic accident	11 (26.2%)
	Assault	7 (16.7%)
	Other	6 (14.3%)

Fracture site	Right	20 (47.6%)
	Left	12 (28.5%)
	Both	10 (23.9%)

Fracture level	Head	6 (11.5%; 4 vertical, 2 horizontal)
	Neck	14 (26.9%)
	Subcondylar	32 (61.6%)

Displacement	Medial override	20 (38.5%)
	Lateral override	18 (34.6%)
	Angulation without override	14 (26.9%)

Dislocation	None	10 (19.2%)
	Slight	12 (23,1%)
	Moderate	6 (11.5%)
	Total	24 (46.2%)

## References

[1] Villareal PM Monje F Junquera LM Mateo J Morillo AJ Mandibular condyle fractures: determinants of treatment and outcome Journal of Oral and Maxillofacial Surgery 2004 62 155 163 10.1016/j.joms.2003.08.010 14762747

[2] Rowe NL Killey HC Fractures of the Facial Skeleton 2nd ed Edinburgh, Scotland E. and S. Livingstone 1968 137 172

[3] Zachariades N Mezitis M Mourouzis C Papadakis D Spanou A Fractures of the mandibular condyle: a review of 466 cases. Literature review, reflections on treatment and proposals Journal of Cranio-Maxillofacial Surgery 2006 34 7 421 432 10.1016/j.jcms.2006.07.854 17055280

[4] Valiati R Ibrahim D Abreu MER Heitz C de Oliveira RB The treatment of condylar fractures: to open or not to open? A critical review of this controversy International Journal of Medical Sciences 2008 5 6 313 318 10.7150/ijms.5.313 18974859 PMC2574020

[5] Lindahl L Condylar fractures of the mandible I: Classification and relation to age, occlusion, and concomitant injuries of the teeth and teeth-supporting structures, and fractures of the mandibular body International Journal of Oral Surgery 1977 6 1 12 21 10.1016/s0300-9785(77)80067-7 402318

[6] Haug RH Brandt MT Closed reduction, open reduction, and endoscopic assistance: current thoughts on the management of mandibular condyle fractures Plastic and Reconstructive Surgery 2007 120 7 Suppl 2 90S 102S 10.1097/01.prs.0000260730.43870.1b 18090732

[7] Brandt MT Haug RH Open versus closed reduction of adult mandibular condyle fractures: a review of the literature regarding the evolution of current thoughts on management Journal of Oral and Maxillofacial Surgery 2003 61 11 1324 1332 10.1016/s0278-2391(03)00735-3 14613090

[8] Ziccardi VB Schneider RE Kummer FJ Wurzburg lag screw plate versus four-hole miniplate for the treatment of condylar process fractures Journal of Oral and Maxillofacial Surgery 1997 55 6 602 609 10.1016/s0278-2391(97)90492-4 9191642

[9] Haug RH Assael LA Outcomes of open versus closed treatment of mandibular fractures Journal of Oral and Maxillofacial Surgery 2001 59 4 370 375 10.1053/joms.2001.21868 11289165

[10] García-Guerrero I Ramírez JM Gómez de Diego R Martínez-González JM Poblador MS Complications in the treatment of mandibular condylar fractures: surgical versus conservative treatment Annals of Anatomy 2018 216 60 68 10.1016/j.aanat.2017.10.007 29223659

[11] House JW Brackmann DE Facial nerve grading system Otolaryngology–Head and Neck Surgery 1985 93 2 146 147 10.1177/019459988509300202 3921901

[12] Helkimo M Studies on function and dysfunction of the masticatory system. II. Index for anamnestic and clinical dysfunction and occlusal state Swedish Dental Journal 1974 67 2 101 121 4524733

[13] Helkimo M Studies on function and dysfunction of the masticatory system. III. Analyses of anamnestic and clinical recordings of dysfunction with the aid of indices Swedish Dental Journal 1974 67 3 165 181 4526188

[14] Baryza MJ Baryza GA The Vancouver Scar Scale: an administration tool and its interrater reliability The Journal of Burn Care and Rehabilitation 1995 16 5 535 538 10.1097/00004630-199509000-00013 8537427

[15] Kommers SC Boffano P Forouzanfar T Consensus or controversy? The classification and treatment decision-making by 491 maxillofacial surgeons from around the world in three cases of a unilateral mandibular condyle fracture Journal of Cranio-Maxillofacial Surgery 2015 43 10 1952 1960 10.1016/j.jcms.2015.08.031 26498515

[16] Durmus Kocaaslan N Karadede Ünal B Çavuş Özkan M Karadede B Çelebiler Ö Comparison of different treatment techniques in the mandibular condyle fracture Ulus Travma Acil Cerrahi Dergisi 2022 28 1 99 106 10.14744/tjtes.2020.94992 PMC10443169 34967439

[17] Brucoli M Boffano P Romeo I Corio C Benech A Management of mandibular condylar fractures in patients with atrophic edentulous mandibles Journal of Stomatology, Oral and Maxillofacial Surgery 2020 121 3 226 232 10.1016/j.jormas.2019.10.004 31655226

[18] Torre DD Burtscher D Widmann G Pichler A Rasse M Surgical treatment of mandibular condyle fractures using the retromandibular anterior transparotid approach and a triangular positioned double miniplate osteosynthesis technique: a clinical and radiographic evaluation of 124 fractures Journal of Cranio-Maxillofacial Surgery 2015 43 6 944 949 10.1016/j.jcms.2015.04.019 26027860

[19] Satishchandran S Umorin M Manhan AJ Abramowicz S Amin D Does the treatment approach for mandibular condyle fractures impact self-perceived quality of life? Journal of Oral Maxillofacial Surgery 2023 81 2 184 193 10.1016/j.joms.2022.10.006 36375512

[20] Ellis E McFadden D Simon P Throckmorton G Surgical complications with open treatment of mandibular condylar process fractures Journal of Oral and Maxillofacial Surgery 2000 58 9 950 958 10.1053/joms.2000.8734 10981974

[21] Rozeboom AVJ Dubois L Bos RRM Spijker R de Lange J Open treatment of condylar fractures via extraoral approaches: a review of complications Journal of Cranio-Maxillofacial Surgery 2018 46 8 1232 1240 10.1016/j.jcms.2018.04.020 29866435

[22] Vesnaver A Open reduction and internal fixation of intra-articular fractures of the mandibular condyle: our first experiences Journal of Oral and Maxillofacial Surgery 2008 66 10 2123 2129 10.1016/j.joms.2008.06.010 18848112

[23] Algan S Kara M Cakmak MA Tan O Cinal H Experiences with a modified preauricular mini incision with subdermally dissection in condylar and subcondylar fractures of the mandible Journal of Cranio-Maxillofacial Surgery 2018 46 4 588 593 10.1016/j.jcms.2018.01.018 29526414

[24] Marwan H Sawatari Y What is the most stable fixation technique for mandibular condyle fracture? Journal of Oral and Maxillofacial Surgery 2019 77 12 2522e1 2522.e12 10.1016/j.joms.2019.07.012 31472103

[25] Handschel J Ruggeberg T Depprich R Schwarz F Meyer U Comparison of various approaches for the treatment of fractures of the mandibular condylar process Journal of Cranio-Maxillofacial Surgery 2012 40 8 e397 e401 10.1016/j.jcms.2012.02.012 22440318

[26] Meyer C Zink S Chatelain B Wilk A Clinical experience with osteosynthesis of subcondylar fractures of the mandible using TCP plates Journal of Cranio-Maxillofacial Surgery 2008 36 5 260 268 10.1016/j.jcms.2008.01.002 18328720

[27] Ellis E Throckmorton GS Treatment of mandibular condylar process fractures: Biological Considerations Journal of Oral and Maxillofacial Surgery 2005 63 1 115 134 10.1016/j.joms.2004.02.019 15635566

[28] Kozakiewicz M Zieliński R Krasowski M Okulski J Forces causing one-millimeter displacement of bone fragments of condylar base fractures of the mandible after fixation by all available plate designs Materials 2019 12 19 3122 10.3390/ma12193122 31557809 PMC6804126

[29] Rai A Comparison of single vs double noncompression miniplates in the management of subcondylar fracture of the mandible Annals of Maxillofacial Surgery 2012 2 2 141 145 10.4103/2231-0746.101339 23482969 PMC3591062

[30] Pappachan B Agrawal R Comparison of two L shaped plate on plate versus single conventional L miniplate in fixation of subcondylar mandibular fractures Journal of Maxillofacial and Oral Surgery 2019 18 3 440 446 10.1007/s12663-018-1154-8 31371888 PMC6639440

[31] Anirudhan A Khalam SA Zachariah RK Evaluation of clinical use of indigenously developed delta plate in management of subcondylar fracture Clinics and Practice 2013 3 2 e28 10.4081/cp.2013.e28 24765516 PMC3981273

[32] Hammer B Schier P Prein J Osteosynthesis of condylar neck fractures: a review of 30 patients British Journal of Oral and Maxillofacial Surgery 1997 35 4 288 291 10.1016/s0266-4356(97)90050-4 9291270

[33] Ellis E Dean J Rigid fixation of mandibular condyle fractures Oral Surgery, Oral Medicine and Oral Pathology 1993 76 1 6 15 10.1016/0030-4220(93)90285-c 8351124

[34] Kumar S Chugh A Kaur A Aparna G Srivastav S Treatment outcome comparison between two 3-dimensional plates (Y-shaped plate versus trapezoidal condylar plate) in management of mandible condylar fracture: a randomized control trial Journal of Maxillofacial and Oral Surgery 2023 22 1 25 32 10.1007/s12663-021-01662-6 36703652 PMC9871142

[35] Aquilina P Parr WCH Chamoli U Wroe S Finite element analysis of patient-specific condyle fracture plates: a preliminary study Craniomaxillofacial Trauma and Reconstruction 2015 8 2 111 116 10.1055/s-0034-1395385 26000081 PMC4428732

[36] Ben Achour A Meißner H Teicher U Haim D Range U Biomechanical evaluation of mandibular condyle fracture osteosynthesis using the rhombic three-dimensional condylar fracture plate Journal of Oral and Maxillofacial Surgery 2019 77 9 1868e1 1868.e15 10.1016/j.joms.2019.04.020 31112678

[37] Closs Ono MC de Morais AD Freitas RDS de Oliveira e Cruz GA Surgical treatment for extracapsular condylar fractures of the mandible The Journal of Craniofacial Surgery 2018 29 5 1312 1315 10.1097/SCS.0000000000004344 29485575

[38] Tandon S Verma V Rashid M Srivastava S Singh AK Is the facial nerve at risk following surgical correction of mandibular condylar fracture: a systematic review and meta-analysis National Journal of Maxillofacial Surgery 2022 13 Suppl 1 1 10 10.4103/njms.njms_481_21 36393942 PMC9651256

[39] Al-Moraissi EA Louvrier A Colletti G Wolford LM Biglioli F Does the surgical approach for treating mandibular condylar fractures affect the rate of seventh cranial nerve injuries? A systematic review and meta-analysis based on a new classification for surgical approaches Journal of Cranio-Maxillofacial Surgery 2018 46 3 398 412 10.1016/j.jcms.2017.10.024 29339001

[40] Felix K Singh M The retromandibular transparotid approach for reduction and internal fixation of mandibular condylar fractures Annals of Maxillofacial Surgery 2020 10 1 168 177 10.4103/ams.ams_193_19 32855935 PMC7433982

[41] Miloro M Endoscopic-assisted repair of subcondylar fractures Oral Surgery, Oral Medicine, Oral Pathology, Oral Radiology and Endodontology 2003 96 4 387 391 10.1016/j.tripleo.2003.07.004 14561961

[42] Spinzia A Patrone R Belli E Dell’Aversana Orabona G Ungari C Open reduction and internal fixation of extracapsular mandibular condyle fractures: a long-term clinical and radiological follow-up of 25 patients BMC Surgery 2014 14 68 10.1186/1471-2482-14-68 25196114 PMC4163058

[43] Marin P Pouliot D Fradet G Facial nerve outcome with a peroperative stimulation threshold under 0.05 mA The Laryngoscope 2011 121 11 2295 2298 10.1002/lary.22359 22020881

[44] Fearmonti R Bond J Erdmann D Levinson H A review of scar scales and scar measuring devices Eplasty 2010 10 e43 20596233 PMC2890387

[45] Kridel RWH Liu ES Techniques for creating inconspicuous face-lift scars: avoiding visible incisions and loss of temporal hair Archives of Facial Plastic Surgery 2003 5 4 325 333 10.1001/archfaci.5.4.325 12873871

[46] Kommers SC van den Bergh B Boffano P Verweij KP Forouzanfar T Dysocclusion after maxillofacial trauma: a 42 year analysis Journal of Cranio-Maxillofacial Surgery 2014 42 7 1083 1086 10.1016/j.jcms.2013.05.013 23849246

[47] Rozeboom A Dubois L Bos R Spijker R de Lange J Open treatment of unilateral mandibular condyle fractures in adults: a systematic review International Journal of Oral and Maxillofacial Surgery 2017 46 10 1257 1266 10.1016/j.ijom.2017.06.018 28732561

